# Microstructural and Mechanical Properties of Calcium-Treated Cactus Pear Mucilage (*Opuntia* spp.), Pectin and Alginate Single-Biopolymer Films

**DOI:** 10.3390/polym15214295

**Published:** 2023-11-01

**Authors:** Brandon Van Rooyen, Maryna De Wit, Gernot Osthoff, Johan Van Niekerk, Arno Hugo

**Affiliations:** 1 Department of Sustainable Food Systems and Development, University of the Free State, Bloemfontein 9300, South Africa; 2 Department of Microbiology and Biochemistry, University of the Free State, Bloemfontein 9300, South Africa; 3 Department of Animal Science, University of the Free State, Bloemfontein 9300, South Africa

**Keywords:** natural packaging, cactus pear mucilage, pectin, alginate, biopolymer films, cross-linked, mechanical properties, *Opuntia ficus-indica*

## Abstract

Pectin and alginate satisfy multiple functional requirements in the food industry, especially relating to natural packaging formulation. The continuous need for economic and environmental benefits has promoted sourcing and investigating alternative biomaterials, such as cactus pear mucilage from the cladodes of *Opuntia* spp., as natural packaging alternatives. The structural and mechanical properties of mucilage, pectin and alginate films developed at a 5% (*w*/*w*) concentration were modified by treating the films with calcium (Ca) in the calcium chloride (CaCl_2_) form. Scanning electron microscopy (SEM) showed the 5% (*w*/*w*) ‘Algerian’ and ‘Morado’ films to display considerable microstructure variation compared to the 5% (*w*/*w*) pectin and alginate films, with calcium treatment of the films influencing homogeneity and film orientation. Treating the alginate films with a 10% (*w*/*w*) stock CaCl_2_ solution significantly increased (*p* < 0.05) the alginate films’ tensile strength (TS) and puncture force (PF) values. Consequently, the alginate films reported significantly higher (*p* < 0.05) film strength (TS and PF) than the pectin + Ca and mucilage + Ca films. The mucilage film’s elasticity was negatively influenced by CaCl_2_, while the pectin and alginate films’ elasticity was positively influenced by calcium treatment. These results suggest that the overall decreased calcium sensitivity and poor mechanical strength displayed by ‘the Algerian’ and ‘Morado’ films would not make them viable replacements for the commercial pectin and alginate films unless alternative applications were found.

## 1. Introduction

The increased demands for naturally biodegradable packaging solutions have resulted in various biopolymers being investigated for their film-forming potential. Biopolymers displaying functional potential, which can be used in the development of biopolymer films, are of growing interest. Given the nature of biopolymer films’ functionality and biocompatibility, they have also been considered for a diverse range of applications other than packaging, specifically relating to medical applications such as intervertebral disc replacement and, consequently, bone regeneration [1,2]. Due to their diverse and desirable functional properties, pectin and alginate have specifically found favourable applications in developing biopolymer films. However, factors that influence the formation of these biopolymer films have been identified, such as the addition of a cross-linker [3,4].

The diverse chemical and physical properties, biodegradability and biocompatibility of pectin have resulted in its use in developing biopolymer films [5]. The chemical composition of different pectins can vary, although about 60–65% of the molecule must be composed of galacturonic acid (GalA), which can display varying degrees of methyl esterification [6,7]. The presence of these charged groups associated with the pectin polymer is predominantly responsible for altering the polymers’ functional properties in the presence of acidic and basic pH environments and by the introduction of charged ions, such as calcium. Although calcium ions have the ability to alter the rheology of a pectin solution, the main consequence of pectin cross-linking is the formation of hydrogels, specifically harnessed in the development of biopolymer films [3,7,8]. Due to pectins’ ability to successfully form biopolymer films, displaying adequate mechanical and barrier properties, multiple authors have investigated the development of pectin biopolymer films for various applications, with and without the addition of a cross-linker [3,9].

Alginate is another polysaccharide used in the development of biopolymer films. The presence of functional groups, consisting of uronic acid, associated with the alginate polymer is of specific importance, as these charged groups directly determine alginate functionality. These functional groups can be modified to alter alginates’ rheological, biochemical and film mechanical properties [9,10,11]. Typically, the functional properties of alginate can be influenced by the presence of cross-linkers, such as calcium and magnesium. Alginate cross-linking is often described by the ‘egg-box’ model, characteristic of the formation of a three-dimensional (3D) network. This model is similar to that used to describe pectin–calcium cross-linking, although differences can be expected between the two mechanisms [6,7,8]. It was reported that alginate–calcium films displayed overall increased mechanical properties regarding tensile strength than pectin–calcium films, indicating that alginate displays increased cross-linking ability due to its structural conformation and chemical composition, ultimately influencing biopolymer films’ physical properties [12]. Bierhalz et al. [13] reported on differences between pectin and alginate biopolymer film micrographs, with alginate films showing a more homogenous and regular morphology than pectin films. Consequently, alginate films were, in some instances, associated with superior mechanical properties [13].

One of the most, if not the most, important properties of biopolymer films is their mechanical properties (physical strength and elasticity). These properties are essential to ensure the protection and maintenance of the structural integrity of food during transportation, storage and handling [3]. Biopolymer films developed from polysaccharides, such as pectin and alginate, require a drying step to imitate pre-formed plastic packaging. However, the drying of these films is generally always associated with films displaying brittle and even fragile properties. Glycerol has been well established as a plasticizer to reduce brittleness and improve the ease of handling of pectin and alginate biopolymer films [14,15]. Kang et al. [16] investigated the TS and %E of pectin biopolymer films. The authors formed films by immersion of pectin films into 5 and 10% CaCl_2_ solutions, acting as a cross-linker. Films were prepared with no addition of calcium, considered the control films. The results confirmed that the films formed using 5% CaCl_2_ showed an increased TS, 198 MPa higher than that of the control films. Furthermore, the 5% CaCl_2_-cross-linked films showed the lowest elongation at a break potential of 2.6% [16]. Badita et al. [17] investigated the influence of calcium, used as a cross-linker, on ‘dry’ alginate biopolymer films’ properties. The authors found that the alginate–calcium films’ properties were considerably influenced by both the cross-linker as well as the concentration of the cross-linker used. It was further confirmed that hydroxyl and carboxylic groups, associated with the alginate polymer chemical structure, were responsible for the hydrogel formation with the addition of calcium, highlighting the benefits of calcium as a cross-linker in biopolymer films formation [17].

Although pectin and alginate have shown success in the development of biopolymer films, consequential high input costs, in addition to the variability and limitations regarding their availability and functionality, have led to the development and investigation of alternative film-forming polymers. The desirable functional properties displayed by the cactus pear mucilage from *Opuntia ficus-indica* have resulted in its investigation as an alternative biopolymeric material in the development of biopolymer films to address certain shortcomings associated with the current polysaccharides films [14,18,19]. Considering the mucilage precipitate from the cactus pear as a functional polymer can prove beneficial because it is often considered an unwanted by-product from cactus pear processing, resulting in favourable cost implications and general ease of availability. 

Although the native mucilage precipitate has been well investigated, variations in its chemical structure and composition have been reported. In general, mucilage is considered a highly flexible heteropolysaccharide with a high molecular weight, which has the potential to carry a negative charge due to the presence of galacturonic acid associated with its chemical structure [20,21,22]. In addition to charged sugars, various amounts of neutral sugars have also been associated with the mucilage precipitate, which includes L-arabinose, D-galactose, L-rhamnose and D-xylose in varying quantities [20,21,22]. Structurally, the mucilage precipitate has been reported to be composed of a charged linear core with many natural sugar side chains. These two main fractions of the mucilage precipitate have been referred to as a gelling, pectin-like fraction and a more neutral, pure mucilage fraction. However, great variability in sugar composition for both fractions has been reported by authors [23,24,25].

In a recent study by van Rooyen et al. [26], the authors showed mucilage, when added at 0.25% and 1.0% to pectin-based composite (blended) films, to enhance certain mechanical properties of pectin films. The authors further suggested these enhanced film mechanical properties could directly be linked to the addition of mucilage to the pectin films [26]. Although this before-mentioned study evaluated mucilages’ compatibility in combination with commercially available polymers, limited knowledge is available on mucilages’ comparative ability to produce single-biopolymer (homopolymeric) films displaying suitable mechanical properties with sights on using them as biodegradable packaging. Logically, comparing single-polymer mucilage films to well-established pectin and alginate films will further advance the understanding of mucilage’s potential to be considered as a homopolymeric film-forming polymer, consequently providing insight into the polymer’s functional potential. The factors that have been shown to alter well-established polymer films’ properties also remain greatly unexplored for single-polymer mucilage-based biopolymer films. Therefore, this study aimed to gain a better understanding of single-polymer (homopolymeric) mucilage films relative to commercially produced single-polymer pectin and alginate films, specifically, to understand the influence a gelling cation (calcium, in the form of calcium chloride) would have on mucilage films’ physical properties so to possibly consider them as alternative biopolymers to pectin and alginate in developing biodegradable packaging. Consequently, exploring the physical properties of these single-polymer mucilage films will contribute to the understanding of the native mucilage precipitate, creating a clearer image of its structural functionality and single-polymer film-forming potential. 

## 2. Materials and Methods

### 2.1. Materials

#### 2.1.1. Commercial Polymers 

Pectin powder with ≤10% moisture content, sourced from apple and sodium alginate powder with both glucuronic and mannuronic acid content, both supplied by Sigma-Aldrich, Cape Town, South Africa, was used. 

#### 2.1.2. Mucilage Precipitate and Freeze-Dried Mucilage Powders

The ‘Algerian’ and ‘Morado’ cultivars of *Opuntia ficus-indica* were used to prepare freeze-dried mucilage powders from a liquid mucilage precipitate with ≤10% moisture content. Following a well-established extraction procedure, described by Du Toit and De Wit [27], which is cost-effective and easily replicated, native mucilage precipitates were prepared. The resultant mucilage precipitates were well characterized in previous studies [28,29]. We used ~24-month-old cactus pear plant cladodes from the ‘Algerian’ and ‘Morado’ cultivars sourced from an orchard (University of the Free State, 29.1076° S, 26.1925° E) with a cactus pear density of 666 cactus pear/h, without irrigation. Firstly, to promote liquid mucilage precipitation, whole cladodes were cubed and microwaved at 900 W for 4 min and then macerated. Secondly, the macerated cladodes were centrifuged using a Beckman^®^ Centrifuge (2315, Brea, CA, USA) at 8000 rpm for 15 min at a constant temperature of 4 °C, which effectively separated the mucilage precipitate from the solids. The liquid mucilage precipitate was then used to prepare the freeze-dried mucilage powders used in this current work. Freeze-drying involved moisture removal from the mucilage precipitate at a low temperature (−30 °C to −40 °C) and constant vacuum until a 95% sample weight loss was obtained. The freeze-dried samples were then milled finely, using a standard mortar and pestle, obtaining a consistent powder with a moisture content of ≤10%. 

#### 2.1.3. Cross-Linker and Plasticizer 

Granular calcium chloride, anhydrous, purchased from Sigma-Aldrich, Cape Town, South Africa, with excellent water solubility, and glycerol (Merck, Johannesburg, South Africa) as a plasticizer, with a purity > 99% were used.

### 2.2. Film Preparation and Development

#### 2.2.1. Film-Forming Solutions 

Using film-forming methods, as described by van Rooyen et al. [26], all film-forming solutions were prepared. The basic method involved dispersing the desired amounts of polymer powder into distilled water containing glycerol. The pectin and alginate films required a minimum of 60% glycerol (*w*/*w*, based on the polymer weight used in the films). The mucilage films required 20% glycerol addition. 

Making use of a magnetic stirrer (Freed Electric-Model MH-4, Rehovot, Israel), all film-forming solutions were allowed to homogenize for 30 min, promoting polymer rehydration at ambient temperature (~25 °C). The rehydrated samples were then further mechanically homogenized by means of a Stick Blender (Mellerwave-Model 85200, Cape Town, South Africa) for 10 s. To reduce the potential volume and structural differences caused by excess entrapped air, the homogenized samples were degassed under vacuum (Genesis Vacuum Sealer, Verimark (Pty) Ltd., Pretoria, South Africa) [26].

#### 2.2.2. Development of Single-Polymer Films

The films used in this study were developed using a standardized batch film casting method with some modifications [5,12,26,30,31]. According to the casting method, the film-forming solution was evenly spread onto a non-stick surface. Upon drying, measurable films formed [5]. Using 140 mm diameter Petri dishes, 70 g of film-forming solution was evenly spread onto the Petri dishes. The dried films were formed by placing the Petri dishes, containing 70 g of film-forming solution, into a ventilated oven (EcoTherm—Model 920, 1000 W, Labotec) at 40 °C for 24 h [26]. Before film evaluation could be completed, all dried films were equilibrated in a closed container at room temperature (~25 °C) for 24 h to achieve ~52% RH.

#### 2.2.3. Calcium Treatment of Single-Polymer Films 

The calcium treatment of the pectin and alginate films required a calcium chloride (CaCl_2_) solution to be directly poured into a polymer solution contained in Petri dishes. After a 5-minute reaction time, the excessive CaCl_2_ solution was removed from the Petri dishes, with the excess CaCl_2_ gently being dabbed off the resultant films with a paper towel [26]. The stock CaCl_2_ solution was prepared at 10% (*w*/*w*) with distilled water; this was considered a more than adequate amount of calcium to react with pectin and alginate [32]. The mucilage films, however, required CaCl_2_ to be directly mixed into the prepared, fully rehydrated, film-forming solution before casting and drying the films. The CaCl_2_ addition was calculated at 30% (*w*/*w*, based on the polymer weight used in the films). Espino-Díaz et al. [18] described similar procedures and calcium concentrations for the calcium treatment of mucilage films. Trachtenberg and Mayer [33] also suggested 30% CaCl_2_ additions to be more than adequate to interact with mucilage. Once the various polymers had been treated with calcium, the films were dried.

### 2.3. Film Characterization 

#### 2.3.1. Scanning Electron Microscopy 

All biopolymer films were subjected to scanning electron microscopy (SEM) imaging. The various samples were first mounted on carbon tape and splutter-coated with iridium. Imaging was then carried out on the various samples at 3.0 KV by making use of a JEOL (JSM-7800F Field Emission, Tokyo, Japan) scanning electron microscope. 

#### 2.3.2. Film Microstructure Evaluation

The images used in this current work were evaluated and selected by a team of five researchers with experience in microscopy, so to represent the overall microstructures of the different images taken of the various films at 10,000× magnification. Additionally, differences observed by the five researchers regarding the differently treated films’ surface roughness/smoothness were also considered by examining visual differences in the surface morphology of the resultant film SEM images.

#### 2.3.3. Mechanical Properties 

Tensile and puncture tests were completed to determine the various films’ mechanical properties using a Texture Analyzer CT3™ (Brookfield AMETEK^®^, Westville, South Africa) with similar methods as those described by van Rooyen et al. [26]. All the film mechanical tests were approached and completed by thoroughly consulting the ASTM international standard methods (ASTM-D882; 2010), as also described by Harper [34]. The various films were cut into 25 × 80 mm rectangular strips, which could then be used to determine their mechanical properties. Twelve films were tested per treatment. The different film thicknesses were measured on all the conditioned films before completing the mechanical tests using a digital micrometre (Grip, South Africa).

##### Film Tensile Test

The Roller Cam Accessory grips (TA-RCA), set at a spacing of 50 mm and a test speed of 0.80 mm·s^−1^, measured the films’ tensile strength and elongation at break values (Brookfield AMETEK^®^, Westville, South Africa). Specifically, a film’s tensile strength is representative of the maximum stress (force/area) any given film can withstand when a force is applied to it [12]. The film tensile strength is specifically calculated by dividing the maximum load (N) by the initial cross-sectional area of a film and is expressed in MPa [14,18,35]. A second tensile test, that is, the elongation at break percentage of a film, specifically measures a film’s maximum capacity to extend before reaching its breaking point [12], calculated by dividing the difference in length of the film at rupture by the initial film length [36].

##### Puncture Test

The various films’ puncture force and distance to puncture were determined by making use of the texture analyzer, accompanied by a Probe TA44 (4 mm diameter probe). A film’s puncture force is defined as the maximum force (N) required to tear the film [37]. All puncture tests were completed by selecting a test speed of 0.80 mm·s^−1^ on the texture analyzer. 

### 2.4. Experimental Design 

The impact CaCl_2_ had on mucilage, pectin and alginate films’ microstructures and mechanical properties was considered. Firstly, using SEM, the various films’ surface microstructures were also investigated. Non-calcium-treated and calcium-treated micrographs were investigated for mucilage, pectin and alginate at 10,000× magnifications. Secondly, the influence CaCl_2_ had on the mucilage, pectin and alginate films’ mechanical properties was evaluated. Due to the calcium sensitivity displayed by the pectin and alginate polymers, CaCl_2_ solutions were prepared and used to cross-link the liquid film-forming pectin and alginate solutions once they had been cast into the Petri dishes. However, Espino-Díaz et al. [18] suggested mucilage’s lower sensitivity to calcium requires CaCl_2_ to be directly mixed into the prepared mucilage film-forming solutions, whereafter film casting, drying, and evaluation could be completed. 

### 2.5. Statistical Analysis

The results of the various trials were captured using Microsoft Excel (2016), and the data were subjected to statistical analysis (ANOVA) using one-way analysis of variance (NCSS Statistical Software package, version 11.0.20). Using the Tukey–Kramer multiple comparison test (α = 0.05), significant differences between the treatment means (NCSS Statistical Software package, version 11.0.20) were identified accordingly.

## 3. Results

### 3.1. Film Microstructure Characterization 

**Figure 1:**
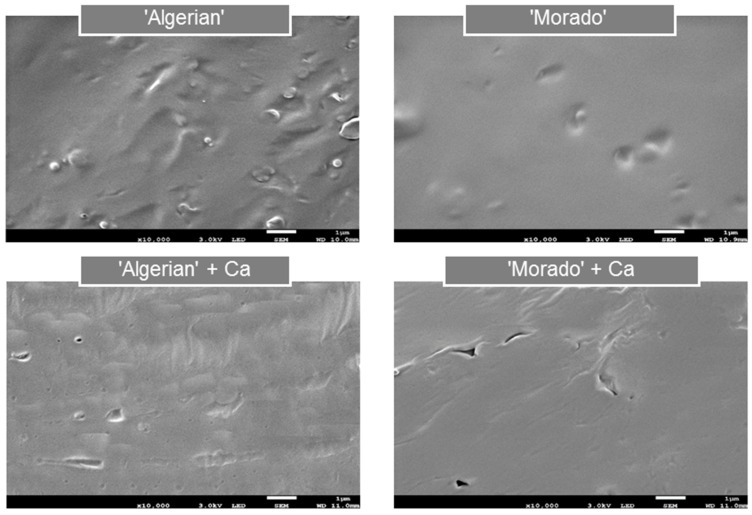
Surface scanning electron microscopy (SEM) images of ‘Algerian’ and ‘Morado’ mucilage ‘dry’ films, with and without calcium (Ca) treatment at 10,000× magnification.

A biopolymer film’s microstructure is often directly associated with its resultant mechanical behaviour [38,39]. Investigating scanning electron microscopy (SEM) micrographs were considered to provide a more holistic insight into the homogeneity and microstructures of non-calcium-treated and calcium-treated mucilage, pectin and alginate films, as calcium is specifically known for its ability to physically alter a film network. Firstly, the surface morphology of ‘Algerian’ and ‘Morado’ mucilage films was considered, as displayed in Figure 1.

The non-calcium-treated ‘Algerian’ films showed a lack of homogeneity, with a surface roughness, compared to the calcium-treated ‘Algerian’ films. The non-calcium-treated ‘Morado’ films showed a smoother, more homogeneous surface morphology than the non-calcium-treated ‘Algerian’ films. However, some cracks and breaks in film homogeneity were observed when treating the ‘Morado’ films with calcium. Guadarrama-Lezama et al. [40] suggested that the appearance of cracks and holes observed in a film microstructure could indicate a denser film formation. The breaks in structure observed in the calcium-treated ‘Morado’ films could thus be related to films of increased density compared to calcium-treated ‘Algerian’ films. Furthermore, treating the mucilage films with calcium showed indications of the development of a more organized mucilage film network (Figure 1). Authors suggested that the presence of a pectin-like fraction that displays sensitivity to calcium could be the reason for the development of these more organized film networks when the mucilage films were treated with calcium [19,23].

**Figure 2:**
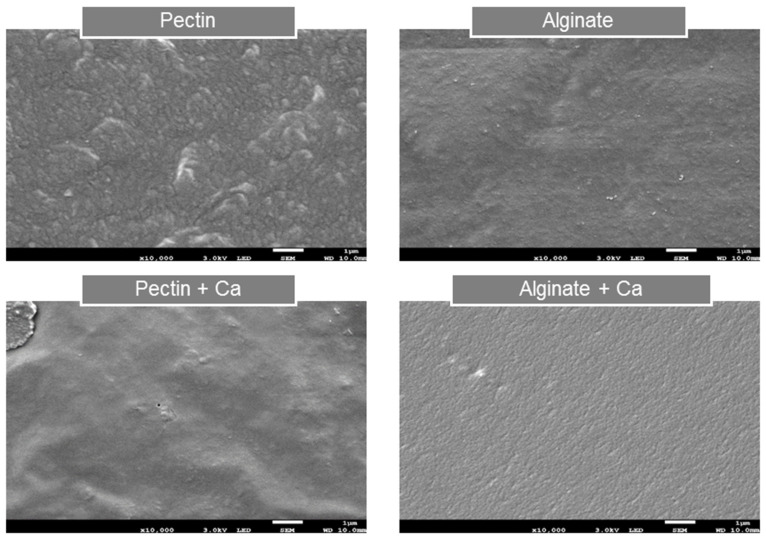
SEM imaging of the surface microstructures of ‘dry’ pectin and alginate films, with and without calcium (Ca) treatments at 10,000× magnification.

The SEM imaging of the surface morphology of the pectin and alginate films, with and without calcium treatment, is presented in Figure 2.

Less homogeneous and more rough surfaces were observed in both the non-calcium-treated pectin and the alginate films’ microstructures compared to their calcium-treated counterparts (Figure 2). Specifically, the calcium treatment was shown to change the surface morphology of the pectin and alginate films noticeably. However, the calcium-treated alginate films showed homogeneity, represented by a more uniform microstructure than the calcium-treated pectin films. This observation indicates the different internal arrangements of the different polymers during film formation, with alginates displaying a more organized network than pectin films [41].

Paşcalau et al. [42] also reported that alginate films treated with calcium resulted in the formation of a more homogeneous film network, typically expected for films that underwent cross-linking (which was not observed for the uncross-linked alginate films), similar to that observed in the current research for uncross-linked and calcium cross-linked alginate films (Figure 2). As observed in the current research, the pectin and alginate films’ microstructures were, therefore, similar to those observed in the literature, with alginate films forming a more organized, homogeneous film network when treated with calcium than pectin films (Figure 2).

The results proved that treating the mucilage, pectin and alginate films with calcium led to noticeable morphological changes in the films’ structures. Microstructure differences were also clearly observed between the mucilage, pectin and alginate films. More homogeneous and organized film networks were found in both pectin and alginate films’ microstructures. In contrast, the mucilage films appeared to be more non-homogeneous and irregular and sometimes contained aggregates and pores. 

### 3.2. Film Mechanical Properties

#### 3.2.1. The Influence of Calcium on Film Thickness 

**Figure 3:**
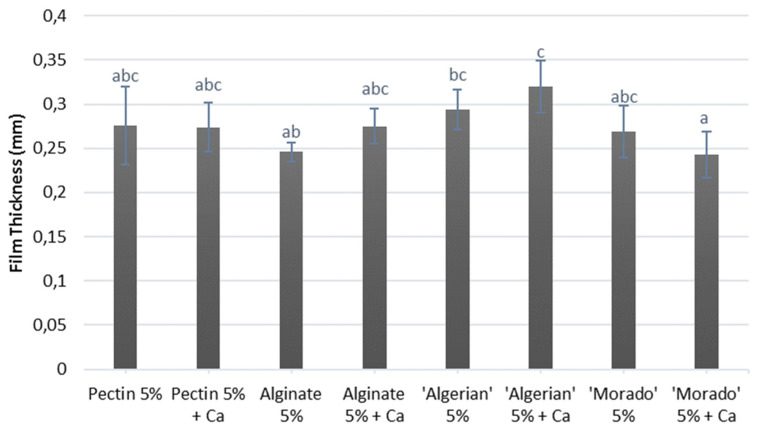
Thickness of the pectin, alginate and mucilage films with and without calcium (Ca) treatment. The mean values of 8 treatments are displayed together with their standard deviations. Error bars represented by differet superscripts differ significantly (*p* < 0.05).

The influence calcium treatments had on mucilage, pectin and alginate films’ thickness is presented in Figure 3. Most films displayed only minimal differences in film thickness. Similar trends in film thickness were reported for films treated with calcium developed at a 5% (*w*/*w*) polymer concentration [26]. However, the ‘Algerian’ + Ca films were significantly thicker (*p* < 0.05) than the ‘Morado’ + Ca films. A decrease in film thickness with the addition of calcium could be related to a higher degree of cross-linking observed in the films due to a possible decrease in inter-chain polymer spacing [9,13]. 

#### 3.2.2. Tensile Test

Further differences were observed between the mechanical properties of the mucilage, pectin and alginate films treated with calcium, as indicated in Table 1. When evaluating the influence the calcium treatment had on the various films’ tensile strength (TS), it was seen that only the alginate film’s TS values were significantly increased (*p* < 0.05) (Table 1). Bierhalz et al. [13] also found alginate films to display superior TS due to the alginate’s linear polymer chains, allowing for a more efficient cross-linking with calcium compared to the cross-linking of the pectin polymer chains. Additionally, alginate also produced films displaying significantly higher (*p* < 0.05) TS values in comparison to the various other films investigated. These findings are also supported by the literature, as authors found alginate to produce films of superior strength due to a more efficient calcium cross-linking of the polymer [12,43,44]. Although calcium-treating the mucilage films showed to have no significant (*p* > 0.05) influence on the film TS, the ‘Morado’ + Ca films did show trends of increased strength, as the TS value increased from 0.31 (TS of the non-calcium-treated ‘Morado’ film) to 1.01 MPa. These findings indicated a slight potential of the ‘Morado’ mucilage to cross-link with calcium.

The film %E showed the calcium treatments to have a considerable and varying influence on film elasticity. The calcium treatments significantly increased (*p* < 0.05) both pectin and alginate films’ %E values whilst significantly decreasing (*p* < 0.05) the ‘Algerian’ and ‘Morado’ mucilage films’ elasticity. It has come to be expected that the addition of calcium would aid in increasing the cohesion forces between the polymer chains of alginate and pectin, increasing film strength and elasticity [13,43]. As regards the decreases in the mucilage film %E values observed in Table 1, research has suggested that, due to the low occurrence of carboxyl groups in the native mucilage structure, the addition of calcium could induce polymer chain contraction or the ionic condensation of the mucilage polymers rather than cross-link the polymer chains, as adequate binding sites for calcium would not be available, in turn reducing the film’s cohesion forces and thus its elasticity [18,23]. The ‘Morado’ + Ca film showed significantly higher (*p* < 0.05) %E values when compared to the ‘Algerian’ + Ca film.

#### 3.2.3. Puncture Test

Similar observations were reported when investigating the various films’ puncture force (PF) values compared to those observed for the films’ TS (Table 2). The calcium treatment of the alginate films significantly increased (*p* < 0.05) the films’ PF values, with the Alginate + Ca films showing the overall highest strength compared to the various other films investigated (Table 2). Again, both ‘Algerian’ and ‘Morado’ mucilage films showed to be only minimally influenced by calcium treatment, producing films with the lowest PF. Interestingly, the puncture tests indicated that the Alginate + Ca films had the highest DTP values compared to the other films. The mucilage films showed the lowest DTP values, with calcium treatment of the films further significantly lowering (*p* < 0.05) them. These trends were similar to those reported for the tensile test %*E* evaluation.

## 4. Discussion

When considering the scanning electron microscopy (SEM) imaging micrographs (Figure 1 and Figure 2) and the mechanical properties (Table 1 and Table 2) of the various films investigated, treating the alginate films with calcium resulted in producing films displaying superior strength compared to the pectin and mucilage films. Guadarrama-Lezama et al. [40] suggested that films with smoother surfaces, lacking pores in the film network, would display superior mechanical properties. Specifically, the calcium-treated alginate films displayed the greatest homogeneity among all the various films investigated. The Alginate + Ca films’ microstructures showed a highly ‘organized’ film network, compared to the calcium-treated pectin films, which were characterized by a less homogeneous surface morphology with visible rough surfaces. Bierhalz et al. [13] also reported that ‘dry’ alginate films’ microstructures presented a more homogenous and regular network than those of ‘dry’ pectin films treated with calcium.

Furthermore, the authors also suggested that alginate’s more linear structure would allow for a more efficient calcium cross-linking than that present in pectin polymers, which consequently would also influence films’ mechanical properties [13]. Therefore, the Pectin + Ca films reported significantly lower (*p* < 0.05) tensile strength (TS) and puncture force (PF) values in comparison to the Alginate + Ca films. Differences in the chemical structures between pectin and alginate have been strongly suggested to be the main reason for the morphological and mechanical differences observed between these two commercially available polymers. As the alginate polymer’s linear chains allow for a more efficient and organized cross-linking, the pectin polymer generally results in a more random orientation with calcium due to its branched chemical structure. Additionally, varying degrees of esterification of the pectin polymer will influence its ability to react with calcium [33,34,35].

When comparing the influence calcium had on the mucilage films, it was seen that both ‘Algerian’ and ‘Morado’ films showed the poorest mechanical properties when compared to the commercially available pectin and alginate films. Authors also reported on calcium-treated mucilage films with low TS and %E values [18]. The lack of homogeneity and the irregular nature of the ‘Algerian’ and ‘Morado’ films must be strongly considered, as these morphological features have been thought to account for films displaying reduced mechanical properties. The expected highly branched chemical structure of mucilage has also been suggested to be strongly associated with the lack of homogeneity and the irregular appearance of mucilage films [19,40]. Furthermore, variations in the surface morphology and mechanical properties were also observed between the two mucilage cultivars. The ‘Morado’ films showed the possibility of increased calcium sensitivity, resulting in films displaying trends of increased strength and elasticity when compared to the ‘Algerian’ films treated with calcium. These findings were supported by examining the microstructural variations between the ‘Algerian’ and the ‘Morado’ films, with less rough film surfaces observed for the ‘Morado’ films. 

Lastly, the ‘Algerian’ + Ca films presented a greater film thickness compared to the ‘Morado’ + Ca films. These findings strongly suggest that the ‘Algerian’ films, being rougher, denser and thicker, would display decreased strength and elasticity compared to the ‘Morado’ mucilage films. Research has suggested that increased film thickness and roughness and decreased film homogeneity could strongly indicate reduced polymer chain interaction in a film matrix, resulting in films displaying reduced strength and elasticity [9,13].

## 5. Conclusions

With global efforts aimed at reducing single-use plastics, the need to develop biopolymer packaging from sustainable sources is being strongly considered. Mucilage, pectin and alginate films displayed varying degrees of mechanical alteration by adding calcium. Although the alginate films showed superior strength, the mucilage films had exceptional elasticity. Cross-linking the various films with calcium showed to enhance further only the alginate films’ tensile strength and puncture strength whilst having only a minimal influence on the pectin and mucilage films’ strength. The superior homogeneity observed in the scanning electron microscopy (SEM) images of the alginate films was clearly identifiable compared to the less organized pectin and mucilage films’ microstructures. The pectin and alginate films’ elasticity was further enhanced by treating the films with calcium, with the mucilage film elongation at break percentage and distance to puncture values negatively influenced by calcium. The various polymers’ diverse interactions with calcium accounted for possible structural variations between the polymers. However, the ‘Algerian’ mucilage polymer showed the likeliness to carry less of a charge than the ‘Morado’ mucilage, showing an overall reduced functionality in this work. Regardless of the mucilage cultivar, the results indicated that the mucilage films were much weaker overall than the pectin and alginate films, although they had, in most instances, superior elasticity. The native mucilage films display commercial viability. Therefore, evaluating the water and gas barrier, crystallization, and anti-fogging properties of these films would be a logical next step in a phased approach to establish a holistic view of cactus pear mucilage to be used as an alternative packaging solution. Regardless thereof, the mechanical properties of these films suggest their success as an alternative, application-specific packaging, likely not finding similar applications as alginate and pectin films.

## Figures and Tables

**Table 1:** Tensile test mechanical properties of non-calcium-treated and calcium-treated mucilage, pectin and alginate films.

Treatments/Films	TensileStrength (MPa)	Elongation at Break %
Pectin 5%	6.41 ± 0.50 ^b^	14.31 ± 1.88 ^cd^
Pectin 5% + Ca	7.01 ± 0.61 ^b^	20.46 ± 2.76 ^ef^
Alginate 5%	17.57 ± 0.90 ^c^	7.79 ± 1.03 ^ab^
Alginate 5% + Ca	20.10 ± 1.07 ^d^	15.68 ± 0.60 ^de^
‘Algerian’ 5%	0.26 ± 0.05 ^a^	33.10 ± 6.10 ^g^
‘Algerian’ 5% + Ca	0.37 ± 0.10 ^a^	4.98 ± 0.67 ^a^
‘Morado’ 5%	0.31 ± 0.10 ^a^	21.58 ± 1.76 ^f^
‘Morado’ 5% + Ca	1.01 ± 0.10 ^a^	10.41 ± 0.70 ^bc^
Significance level	*p* < 0.005	*p* < 0.005

The mean values of 8 treatments, together with their standard deviations (±), are presented. The mean values with different superscripts in the same column differ significantly (*p* < 0.05).

**Table 2:** Puncture test mechanical properties of non-calcium-treated and calcium-treated mucilage, pectin and alginate films.

Treatments/Films	Puncture Force (N)	Distance to Puncture (mm)
Pectin 5%	31.75 ± 2.38 ^b^	4.04 ± 0.38 ^b^
Pectin 5% + Ca	35.94 ± 4.32 ^b^	4.60 ± 0.38 ^b^
Alginate 5%	72.17 ± 4.68 ^c^	5.61 ± 0.37 ^cd^
Alginate 5% + Ca	83.30 ± 5.81 ^d^	6.26 ± 0.54 ^d^
‘Algerian’ 5%	2.43 ± 0.26 ^a^	4.24 ± 0.63 ^b^
‘Algerian’ 5% + Ca	2.38 ± 0.39 ^a^	1.89 ± 0.33 ^a^
‘Morado’ 5%	1.82 ± 0.21 ^a^	4.71 ± 0.89 ^bc^
‘Morado’ 5% + Ca	5.43 ± 0.58 ^a^	2.60 ± 0.47 ^a^
Significance level	*p* < 0.005	*p* < 0.005

The mean values of 8 treatments, together with their standard deviations (±), are presented. The mean values with different superscripts in the same column differ significantly (*p* < 0.05).

## Data Availability

Not applicable.
